# Primary Path Reservation Using Enhanced Slot Assignment in TDMA for Session Admission

**DOI:** 10.1155/2015/405974

**Published:** 2015-03-22

**Authors:** Suresh Koneri Chandrasekaran, Prakash Savarimuthu, Priya Andi Elumalai, Kathirvel Ayyaswamy

**Affiliations:** ^1^Department of Computer Science and Engineering, Tagore Engineering College, Chennai 600127, India; ^2^Department of Electronics and Communication Engineering, Jerusalem College of Engineering, Chennai 600100, India; ^3^HCL Technology, Chennai, India; ^4^Department of Computer Science and Engineering, Anand Institute of Higher Engineering and Technology, Chennai, India

## Abstract

Mobile ad hoc networks (MANET) is a self-organized collection of nodes that communicates without any infrastructure. Providing quality of service (QoS) in such networks is a competitive task due to unreliable wireless link, mobility, lack of centralized coordination, and channel contention. The success of many real time applications is purely based on the QoS, which can be achieved by quality aware routing (QAR) and admission control (AC). Recently proposed QoS mechanisms do focus completely on either reservation or admission control but are not better enough. In MANET, high mobility causes frequent path break due to the fact that every time the source node must find the route. In such cases the QoS session is affected. To admit a QoS session, admission control protocols must ensure the bandwidth of the relaying path before transmission starts; reservation of such bandwidth noticeably improves the admission control performance. Many TDMA based reservation mechanisms are proposed but need some improvement over slot reservation procedures. In order to overcome this specific issue, we propose a framework—PRAC (primary path reservation admission control protocol), which achieves improved QoS by making use of backup route combined with resource reservation. A network topology has been simulated and our approach proves to be a mechanism that admits the session effectively.

## 1. Introduction 

The strength of MANET [1] lies in its ability to form self-organized network which seems to be an interesting fact. A MANET provides a practical way to rapidly build a decentralized communication network in areas where there is no existing infrastructure or where temporary connectivity is needed. This property makes these networks highly flexible. There exist some practical design issues in MANET such as limited bandwidth, dynamic nature of topology, and decentralized coordination. Due to these design issues, the quality factors like bandwidth, delay, and jitter [2] get affected. Certain real time applications such as audio and video expect additional bandwidth in order to provide QoS [3–5]. The goal of QoS is to achieve more deterministic network behaviour so that the information carried by the network can be better delivered and the resources can be better utilized. To provide QoS, we need certain frameworks such as reservation, scheduling, admission control, and routing algorithms [6–9].

Admission control can avoid the network congestion by estimating whether the new session is admissible or not [10, 11]. Various admission control methods have been proposed in many articles in last few years. However the admission control based protocols have certain pitfalls such as improper handling of session. Different mechanism has been proposed for session admission, of which backup mechanism has a noticeable achievement [12, 13]. Admission control algorithm combined with backup path solves the frequent path beak due to mobility; even such mechanism is not utilized properly. The reservation mechanism ensures the QoS [8, 14]. There are many reservation based protocols, such as hop reservation multiple accesses (HRMA) [15] and five-phase reservation protocol (FPRP) [16, 17]. In addition, contention based protocols, such as carrier sensing multiple accesses with collision avoidance (CSMA/CA), and hybrid protocols, such as TDMA protocol based on contention and reservation [18], exist. The reservation based TDMA protocols have several advantages over contention based protocols, of which conflict free and maximum spatial reuse efficiency is acquainted to the domain [14, 19, 20]. For network with dynamic topology FPRP is a suitable one. Even though the performance aspect of FPRP is improved, still the time slot utilization was not improved.

Considering all the issues of admission control, reservation and backup path, we propose a QoS framework named primary path reservation admission control (PRAC). PRAC framework focuses on admission control and reservation, in order to achieve quality of service. Using backup route aided admission control protocols, such as StAC [13], MACMAN [12], introduces too many control overheads, whereas our proposed protocol minimizes the control packets. In the reservation mechanism such as TDMA, the slot assignment procedure for any request does not consider the neighboring slot assignment. Due to the fact that bandwidth is commonly shared by all carrier sensing nodes, it affects the current node transmission. So it is mandatory to consider the neighboring slot assignment when calculating the bandwidth, which further tries to avoid collisions. By using PRAC, we minimize the delay and maximize the throughput for any admitted session, thereby increasing the overall performance.

## 2. Background and Related Works

### 2.1. Admission Control

The admission control (AC) mechanism provides a way that should ensure admissible path from one node to another. Such mechanism determines admission of new data flows by keeping track of available bandwidth; it determines the residual capacity of any individual node and also of the carrier sensing neighbor (*n*_cs_) nodes [10]. The AC Protocol starts with calculation of local nodes capacity. The local capacity of any node can be estimated by any of the quality aware routing (QAR) protocols [21–23]. This protocol discovers the routes which have sufficient resource that satisfies the requirements of a session. Apart from these protocols, the basic routing protocols like DSR [21] can also be used to estimate the local capacity (bandwidth), where the local capacity is the unconsumed bandwidth at a given node. Various methods have been proposed to calculate the local capacity in [11–13], among which channel ideal time (CIT) is suitable for admission control and can be calculated using channel ideal time and link transmission in use [12, 13]. The residual capacity calculation alone does not serve the need of admission control, so the second phase continues with ensuring the adequate capacity of the *n*_cs_. This adequate capacity can be calculated by several methods. In [10], the first proposed method, referred to as CACP-Multihop, gathers information about the residual capacities can be obtained from the neighbors using admission request packets which are flooded through the network with a radius of two hops. The second proposed method, referred to as CACP-Power, uses a high power transmission mechanism to send the admission request packet to all nodes within the *n*_cs_. The other proposed method in adaptive admission control [7] and SoftMAC [11] sends hello packets to calculate the CIT value.

### 2.2. Related Works

We have analyzed a wide range of protocols before landing up in a concept to provide a new way of admission control. The works done by various authors depict that the core concentration is on providing QoS and to address the issues related to them. In-depth analysis of [7, 24] paves way to get a clear picture classifying the admission control protocols and these proposals provide many useful information pieces for forming a more efficient protocol.

In [13], multipath admission control (MACMAN) was proposed. The paper discussed the priority given to QoS by maintaining backup routes, which thereby enhances the performance (by trying to avoid path or packet loss) of the overall network. Even though this method proves optimal in increasing the performance, the concealed fact is that maintaining the backup routes increases control overhead too. An improved version of MACMAN staggered backup (StAC-backup) [13] proposes a technique that involves only partial disjoint set for admission control which reduces the path identification control overhead when compared to other protocols discussed. As the control overhead due to the beacon messages increases, the network data transfer rate decreases. In [25], distributed admission control protocol (DACP) was proposed. This paper contributes mechanisms to provide QoS by making use of bandwidth reservation. In order to reserve bandwidth, DACP estimates local and neighbour capacity. So mentioned protocol does not consider the hidden and exposed terminal problem since reservation of bandwidth may not be accurate and also the paper does not consider backup route based routing.

In [8], an on-demand bandwidth reservation QoS routing protocol for mobile ad hoc networks was proposed, which considers revising the bandwidth estimation and reservation procedure. For route discovery, an approach min-max was used to satisfy the bandwidth requirement. Also the reservation procedure used estimates the weight of its neighbor's slot for availability. The time slot with lowest weight will be reserved. The proposed method admission control and bandwidth reservation (ACBR) in [26] suffers from a limitation that estimates the available capacity of the neighboring nodes, using one-hop distance only. In addition, it does not take the contenting nodes in the interference range into account. In other words, this scheme considers only the contention of nodes within transmission range.

In [18], the proposed TMMR protocol performs bandwidth reservation in order to attain multihop packet transmissions. It also provisions the node mobility through fast fault node detection. TDMA based reservation mechanism is exploited but still it does not improve time slot selection for reservation. The novel idea of time slot utilization proposed in [20] does not consider the impact of hidden terminal problem and it lacks effectiveness in session admission. Considering the above discussed facts, our proposed method exhibits the flow of admission in an efficient reservation, combined with a backup path mechanism. The effect of such an improvised mechanism shows enhanced performance.

## 3. Primary Path Reservation Admission Control Protocol (PRAC)

### 3.1. Backup Route Discovery

Studies on backup route discovery emphasize that many backup routes have to be maintained to overcome re-routing process after a route failure. The disadvantage of the above stated method is that since many control packets are sent through and forth, there is a consistent increase in the network overhead. In PRAC, we maintain a primary path and a single backup path. From previous analysis, dynamic source routing (DSR) [21] proves to be a suitable routing protocol for finding backup routes. The backup route discovery process finds the route in which the nodes involved in the process are in a complete disjoint set. The backup path discovered should not include the nodes that were in the primary path [12]. Results from [13] which include many backup routes prove that the nodes in primary and backup routes can be sufficiently disjoint and are not required to be in full disjoint sets. Consider

(1) Rprimary∩Rbackup≤Rprimary2,

where *R*_primary_ is the primary path and *R*_backup_ is the backup path. But in PRAC since only one backup route is maintained, the backup route nodes should be a complete disjoint set with that of the primary route nodes:

(2) Rprimary∩Rbackup  =  0.

**Figure 1:**
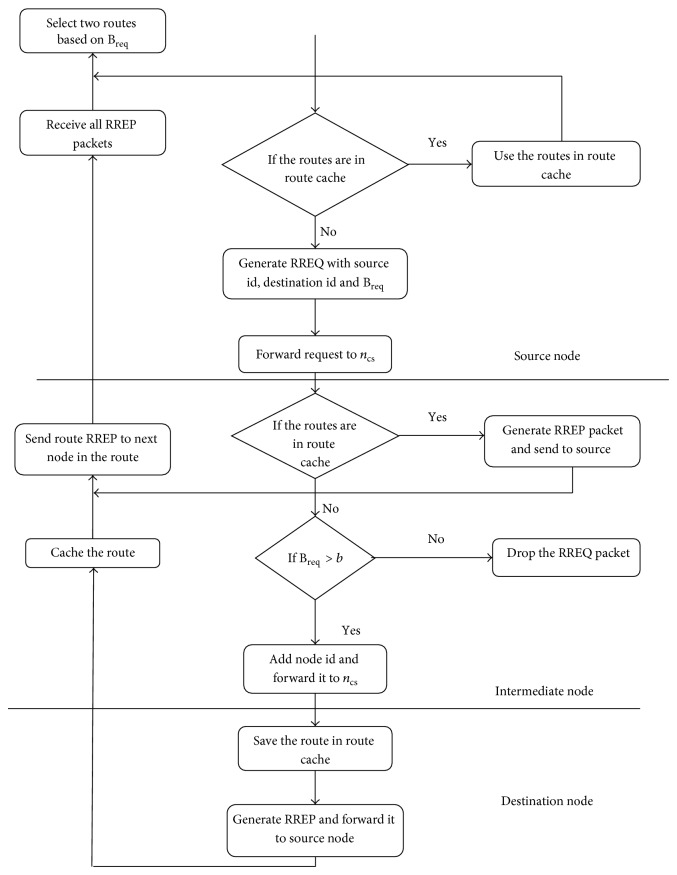
Capacity constraint route discovering process.

If many backup paths are maintained, the condition for partial or sufficient disjoint sets may yield better performance, whereas, in the case of a single backup, if the primary path fails then the probability of backup failure is also feasible. Hence the backup path nodes should be a complete disjoint set, thereby reducing the risk of failure. The capacity constraint route discovering process is explained in Figure 1. The route discovery process starts with the request for session. In this, the source node broadcasts the RREQ packet to all its neighbouring nodes. Each node calculates its own residual capacity, which if satisfies the capacity requested by a session, will rebroadcast the RREQ packet to their neighbours. The session capacity requirement BW_req_ can be calculated as follows:

(3) BWreq=b∗ncs.

This equation is used to calculate the session capacity requirement at any node. Here *b* is the number of slots required and *n*_cs_ is the number of contenting nodes in the carrier sensing range. MACMAN [12] implements high power method that builds up *n*_cs_ sets, which in turn increases the beacon overhead making it a probable disadvantage. Our proposed PRAC method overcomes it by the use of admission request packet (similar to CACP-Multihop), flooded through the network, covering a two-hop radius at the time of session admission, depending upon the session capacity; a node may confirm the session admission or deny it. In the case of primary route failure, backup route discovery is initiated, in which the process is decoupled from the normal route discovery mechanism and only admission control mechanism takes place.

### 3.2. Validation of Single Backup Path over Multiple Backup Paths

Though multiple backup path approach does make sure the backup route availability and better manages the throughput drop, it adds additional overhead in the network by sending probe packet to manage the paths. Moreover the single backup path provides a better way in managing the overall traffic in a given network by avoiding unintended interference that happened on the session routes. As there is only one backup path, the maintenance quotient is virtually lower compared to the multiple backup method (that periodically engages the routes for the probing). Though the problem of handling the mobility/path-break appears to exist in this single backup path, it provides a way to find out another path if initial backup path fails to establish the connection. So in any eventual case the single backup path does make sure that at any point of time there is a path existing for the data transmission. We assume *h* is the number of hops between the source and the destination. The single paths can achieve reduced probe packet as demonstrated in shown calculation below. The analytical verification and the robustness of the single backup path are based on the simple routing model. The probability of packet control overheads denoted *P*_*c*_ between the source and the destination in the single backup path routing:

(4) Pc=1−1  −  μh,

where *μ* is average control packet and *h* is the number of hops. Now we can find that the probability of control packets in multiple backup paths maintained is

(5) Pc=  1−1  −  μhm,

where *m* is the number of disjoint-backup paths. As the number of hops, *h*, and the multiple backup paths, *m* increases the probability of the overhead in the network increases as depicted in the formula (5), whereas in single backup path, the only parameter that causes the overhead to increase is the number of hops and similarly the number of control packets to send also increases. This does make sure that the network traffic is managed better.

### 3.3. Reservation

**Figure 2:**
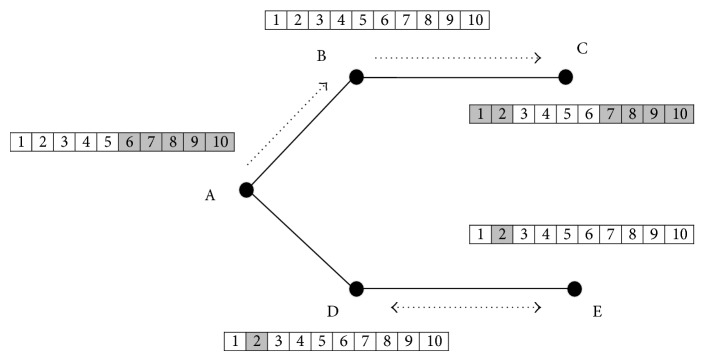
Bandwidth calculation interfered by hidden and exposed terminal problem.

The purpose of backup route discovery is to find the residual capacity of each and every node in a route. Such a mechanism discovers many routes, among which the efficient routes are selected (the mechanism involved in backup route discovery has been discussed in Section 3.1). The reservation approach in our proposal focuses only on the primary route. When the destination node receives the first RREQ packet of a session, it is considered as the primary path and reservation reply is sent along the reverse direction of the same primary path. When the second RREQ packet is received, this path is not reserved but it will be considered as a backup path thereby sending a normal reply. This reservation process is coupled with backup route discovery. The above facts discussed address how the reservation process integrates the route discovery stage. The reason behind using TDMA for reservation in this paper is that it tolerates the radio interference problem and it holds good in our scenario, since all the nodes share the single common channel. We propose a new approach in TDMA which considers both hidden terminal and exposed terminal problem. A glance on the following example helps us to understand the problem in making reservation. In Figure 2, consider the path from A to C. Here the grey slots depict that they are busy and similarly the white slots are free. Between A and B there are five matching free slots {1, 2, 3, 4, 5} and between B and C there are four matching free slots {3, 4, 5, 6}. If we reserve slots {1, 2, 3} for A to transmit and slot {4, 5, 6} for B to transmit, then the path bandwidth is only three. Suppose that if the other pair D and E are currently using slot 2 to communicate, two possible cases will arise.

**Case 1 .**

If D is the receiver on slot 2, then A will not allow sending on slot 2, so collision occurs at D. This is called hidden terminal problem since common free slots between A and B are reduced to {1, 3, 4, 5}.

**Case 2 .**

Due to Case 1, the bandwidth of the path A to C degrades to 2 slots. If D is the sender on slot 2 then A will not allow sending on slot 2. This is called exposed terminal problem.

#### 3.3.1. System Model

1. Each node maintains the neighbor's information (including available slot). This information is gathered by hello message.
2. TDMA frames are fixed length; time frame had 16 time slots, with 5 ms for each time slot.
3. Nodes shared single common channel.
4. Each frame consists of two subframes: the data frame and control frame. The data frame consists of fixed number of data slots. When a node wants to transmit or receive data packets, it may use its control slot to reserve the desired data slots. In the control frame, each node has a committed control slot and therefore avoids contention and collision.
5. Control and data slot handshake: the control slot holds control packets such as the route request, hello packet, the route reply, and the route error. When a node wants to transfer data it may use the control packets to reserve the required path. Once it is reserved, it can be used for both transmitting and receiving data.

#### 3.3.2. Data Structure

- We maintain sending slot table (*SS*), receiving slot table (*RS*), and hop count table (*H*) in every node. The sending slot table *SS*_*X*_[1 ⋯ *n*, 1 ⋯ *s*]: of node *X* records the time slots of all the nodes within 2 hops from *X* and is having sending activities. So *SS*_*X*_[*i*, *j*] = 1 if slot *j* of node *i* has been reserved for transmission; otherwise, *SS*_*X*_[*i*, *j*] = 0. The receiving slot table *RS*_*X*_[1 ⋯ *n*, 1 ⋯ *s*]: of node *X* records the time slots of all the nodes which are within 2 hops from node *X* and are having receiving activities. Similarly, *RS*_*X*_[*i*, *j*] = 1 if slot *j* of node *i* has been reserved for receiving; otherwise, *R*_*X*_[*i*, *j*] = 0. The hop count table *H*_*X*_[1 ⋯ *n*, 1 ⋯ *n*]: of a node *X* maintains a record of the mutual distances between nodes in *X*'s neighborhood. Similarly, for each node *i* that is within 1 hop from *X*; *H*_*X*_[*i*, *j*] = 1 if node *j* is within 1 hop from *i*; otherwise, *H*_*X*_[*i*, *j*] = *∞*.
- The RREQ has following parameters: RREQ(*S*, *D*, *id*, *X*, *b*, *path*, *NH*) where *S* is source node and *D* is the destination node and *id* is the session identity issued by source; *b* is bandwidth requirement, which can be represented by the number of slots. *X* is the node that is currently relaying RREQ. The *path* is the partial path, with the available slot, that has been discovered so far. It has the format ((*h*_1_, *l*_1_), (*h*_2_, *l*_2_),…, (*h*_*k*_, *l*_*k*_)). Here *h*_*i*_ is node identity, where *i* = 1,…, *k* and each *l*_*i*_ contains the total *b* slots that are found to be available for *h*_*i*_ to transmit to *h*_*i*+1_. *NH* is neighbour hop list ((*h*_1_′, *l*_1_′), (*h*_2_′, *l*_2_′),…). Here each node *h*_*i*_′ may serve as the next hop of node *X* that extends the current partial path, only if *h*_*i*_′ has sufficient slots and also *l*_*i*_′ contains *b* slots that can be used by *X* to transmit to *h*_*i*_′.
- The RREP has following parameters: RREP(*S*, *D*, *id*, *path*). When a route is found at the destination *D*, we need to initiate a packet RREP to the source node *S*. This packet traverses through the reverse path and reserves the slots on the *path*.

**Lemma 1 .**

A slot *t* can be used by a node *X* to send to another node *Y* without causing collision, if the following conditions are satisfied.

1. Slot *t* is not scheduled to send/receive node in neither *X* nor *Y*.
2. For any 1-hop neighbor *Z* of *X*, slot *t* is not scheduled to receive data in *Z*.
3. For any 1-hop neighbor *Z* or *Y*, slot *t* is not scheduled to send data in *Z*.

#### 3.3.3. Reservation Procedure

*(A) Route Request Phase*. When a node *Y* receives a broadcast packet RREQ(*S*, *D*, *id*, *b*, *X*, *path*, *NH*) from a neighbour node *X* and if *Y* has not received the same packet before, then Algorithm 1 will be executed. As shown in Step 1, if *NH* does not have the node *Y* listed in it, then a *path*_*temp* will be created. Hence the corresponding parameter *l*_*i*_′ that contains *b* time slots can be used by *X* to transmit to *h*_*i*_′ without collision. In Step 2, the temporary tables for sending and receiving *SS*_*temp* and *RS*_*temp* are used during the probing stage, in which the confirmed list is stored in original *SS* and *RS*. As the path is propagated for each slot of a node, the confirmed slot information is saved in the respective temporary tables *SS*_*temp* and *RS*_*temp*. This confirmed slot in respective node is carried over to next node that is visited and this will continue till the destination node.

(1) When RREQ arrives at intermediate nodes (see Algorithm 1), in Step 3 the information of *path* and *NH* will be saved in the temporary tables that we have discussed in Step 2. The *slot*_*selection*(*Y*, *Z*, *b*, *SS*_*temp*, *RS*_*temp*) routine is called to check if there is any slot available for *Y* to send *Z*. In case of any slot available in one hop to extend the current path, then the RREQ will be rebroadcasted. The same routine is used for node *Y* to choose the free time slots *b* in order to send data to *Z*. The slot selection procedure is based on Lemma 1. In case, if the required free slot is available, then the algorithm will proceed further to know if there are more free slots to occupy. This is mainly to increase the channel reuse which is essential in the case of wireless communication. Selection of these free slots is done, giving high priority to the ones (node) without the hidden and exposed terminal problems. To achieve this, we give the valid time slot *i*, an increased priority so that *SS*_*temp*[*W*, *i*] = 1 to the neighbour *W* of *Z*.

(2) When RREQ arrives at destination node, a confirmed path is formed once the destination *D* receives the RREQ(*S*, *D*, *id*, *b*, *X*, *path*, *NH*). The destination node *D* can still accept the request RREQ or choose ignoring it, based on following condition. When a node *D* receives a broadcast packet RREQ from a node *X*, then if *NH* does not have *D* listed in it, then a *path*_*temp* will be created. The corresponding parameter *l*_*i*_′ contains *b* time slots that can be used by *X* to transmit to *h*_*i*_′ without collision. Then RREP will be sent to the source *S*.

- Let (*h*_*i*_′, *l*_*i*_′) be the entry in *NH* such that *h*_*i*_′ = *D*.
- *path*_*temp* = *path*∣(*X*, *l*_*i*_′).
- Send RREP(*S*, *D*, *id*, *path*_*temp*) to source *S*.

*(B) Route Reply Phase*. As a part of the reply sequence, the *RREP* packet will be sent in the reverse direction of *path* in unicast manner with each intermediate node relaying the packet. Also the “receive” and “send” information will be saved in the respective table in each node where the packet is traversed. Assuming that *path* contains ((*h*_1_, *l*_1_), (*h*_2_, *l*_2_),…, (*h*_*k*_, *l*_*k*_)), each intermediate node will update the sending and receiving table (confirmed one) with available time slot information (see Algorithm 2).

### 3.4. Route Maintenance

**Figure 3:**
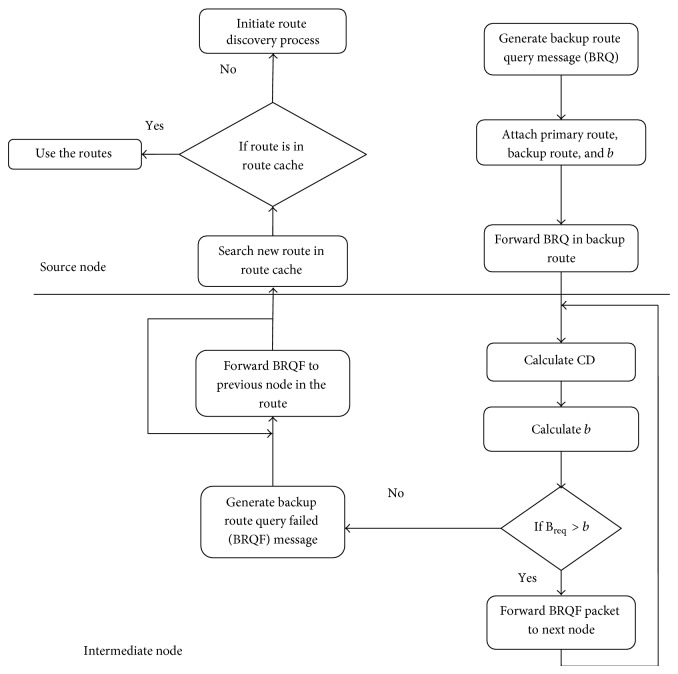
Route maintenance.

The fact behind route maintenance is that the process monitors the primary and backup routes on a regular basis, checking for QoS requirements (session requirements) [12, 13]. In our proposed method, when the primary route fails, the control moves towards the backup path. Here, PRAC makes use of BRQ (backup route query) message that continuously monitors the stability and effectiveness of the backup path. Previous studies show that many other mechanisms use multiple backup routes, which accounts for increase in reliability but also increases the control overhead. But in our model, the possibility of the primary path failure is very low because of the reservation mechanism used, as described before. The control overhead in PRAC model is very low because path monitoring does not comprise many backup paths and instead is done for a single backup path only. In Figure 3 we describe overall route maintenance process. In case of failure of the primary path, in order to maintain the reliability of the network, the backup path is taken. This backup path is herewith considered to be primary and starts reservation process. Since our proposed model uses a single backup strategy, when the backup is taken as primary (in case of failure), then we lack the existence of a backup path (PRAC requires the presence of a primary and backup path always). Hence the source finds a backup path from route cache. As mentioned before, with the use of BRQ message, the route cache is analysed for the best route that accomplishes the session request. When a node receives a BRQ message, it calculates the contention difference (CD). In our maintenance model, we avoid the calculation of contention count. The reason is that the transmission flow along the primary path is likely to reduce the measured available bandwidth along the backup path. Calculating contention count may end up in insufficient metric; hence the contention difference proves to be an optimal method:

(6) CD=CSneighbor∩Rbackup−CSneighbor∩Rprimary.

The CD should hold a constraint in which BW_avail_ > CD · BW_req_. If the above condition fails, then it sends a BRQF message to the source, thereby removing the specific route from route cache. The advantage of PRAC route maintenance is that in the case of both primary and backup path being failed, the source does not go for route recovery process again and instead it makes use of the route cache information [21].

## 4. Performance Evaluation

### 4.1. Simulation Environments

We use NS-2 network simulator to verify the PRAC's performance. Based on our analysis, set of simulations involves a larger network with random mobility. Following are the specifications that have been followed while simulating the PRAC protocol. 100 nodes are randomly placed in a 1000 m × 1000 m area. By setting the node transmission range of 250 m and a CS-Range of 550 m, multihop routes have been created and also allowed all types of collisions to occur. 50 nodes are randomly chosen as sources of traffic to 50 other nodes. Each session was CBR traffic flows that were used with a packet size of 512 bytes and a bit rate of 128 kbps. The nodes move according to the random waypoint mobility model and the bandwidth of the channel is 2 Mbps. The two ray ground propagation model was employed to avoid wireless channel errors. Backup route query (BRQ) interval is 2 s (Table 1).

### 4.2. Results and Discussions

**Figure 4:**
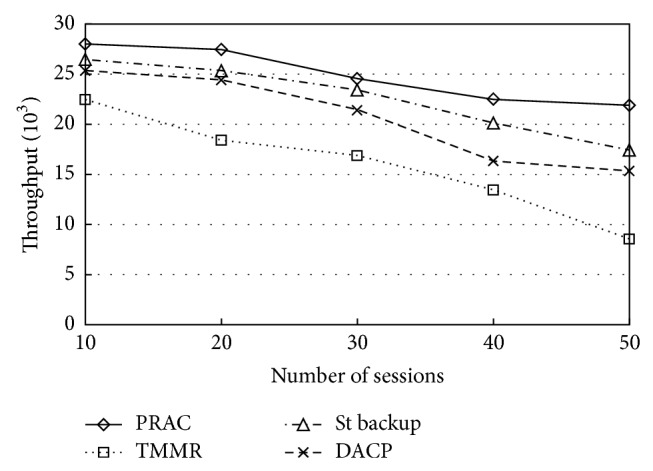
Throughput.

Figure 4 shows the throughput of each session attained by PRAC. In the results, the number of admitted sessions is more or less similar; in addition, the throughput of the admitted session is higher than other models during the simulation time. As shown, we obtain higher throughput while using PRAC. However, when St backup [13] and DACP [25] are used, only medium throughput is obtained and when TMMR [18] is used, only lower throughput is obtained. From the results it is clear that the proposed protocol is capable of reducing the number of unnecessary routing packets during route discovery, by making admission control decisions at every node in the network. Thus, DACP can use more resources in the network than other models to transmit data packets. The admission control without backup path (i.e., TMMR and DACP) model shows poor throughput, while the admission control with backup path (St backup and PRAC) shows high throughput. In addition, PRAC attains greater aggregated throughput than other models.

**Figure 5:**
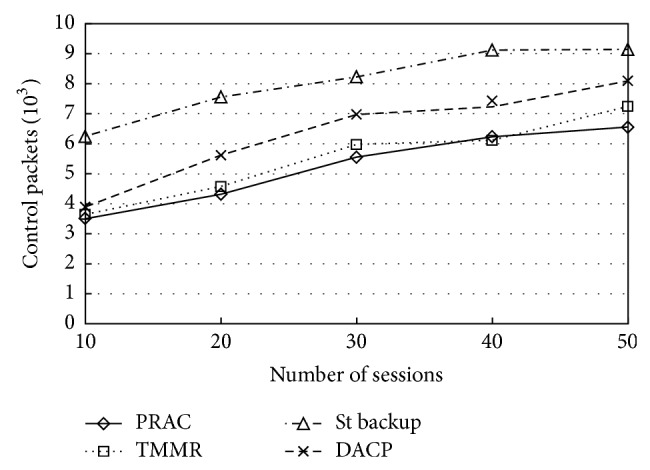
Control packets overheads.

It is also essential to inspect the overhead produced by the protocol since PRAC introduces additional control messages.  Figure 5 demonstrates the average amount of control packets transmitted for the duration in the simulations. As expected, the graph demonstrations a notable increase in control traffic in the network when we use backup path. When compared to the existing protocols our PRAC protocol noticeably produces lesser control packets. As seen in Section 3.3, the actual amount of control packet overhead is an important factor for maintaining a single backup path. A tradeoff happens between the extra control packet overhead and the facility to quickly switch to a new path if the present one breaks down. However, there is an increase in overhead if the protocol uses backup routes that are to be maintained. But this increase is acceptable due to the improvement of the other performance parameters. At first, the overhead is low; this is because of minimum number of sessions that are admitted and also the backup route maintenance messages are produced on a per-flow basis for admitted flows.

**Figure 6:**
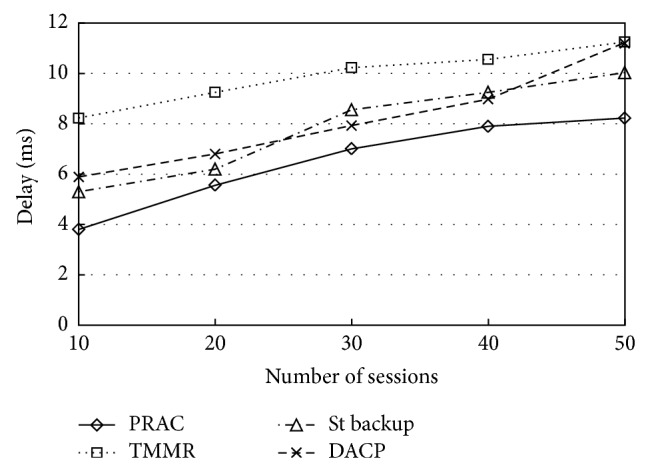
End to end delay.

Since any admission control protocols are mainly designed to put up real time applications that have requirements on end-to-end delay, it is essential to make sure that the additional overhead does not include added delay that go above the delay bounds of the requests [27]. The end-to-end delay for the sessions can be seen in Figure 6. From this figure, PRAC is capable of providing an increase in data packet delivery and minimizing the end-to-end delay. Since there is no admission control performed in TMMR, the network becomes congested as new sessions are added to the network, resulting in decreased throughput and dramatically increasing the delay of the sessions. On the other hand, the throughput of the sessions shows significant degradation; also the delay rises as the number of sessions increases. The average end-to-end delay in the simulations that is achieved for all other three protocols is much higher than PRAC, indicating PRAC's ability to balance the network load.

**Figure 7:**
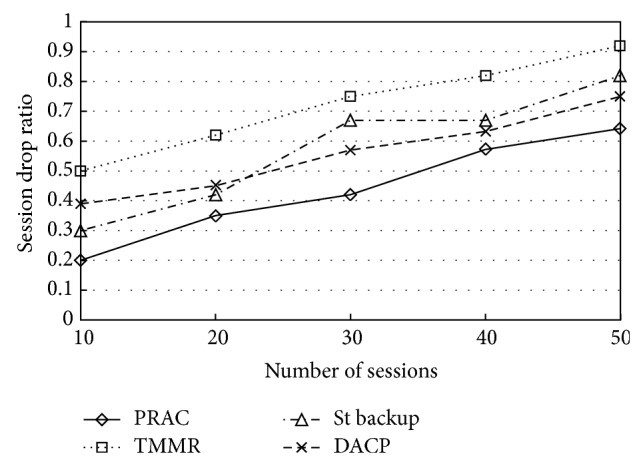
Session drop ratio.

Figure 7 shows the average number of times the sessions are stopped per simulation. When using TMMR, DACP, and St backup, a large number of sessions are stopped. When using PRAC, in many cases, these same sessions can switch to a backup route and retain the transmission. For all the other protocols, it is likely that as the number of admitted sessions increases so does the number of session breaks. However, PRAC is able to handle the session breaks even if large amount of session is admitted. The reduced number of session drops is likely to increase the QoS at the end user.

**Figure 8:**
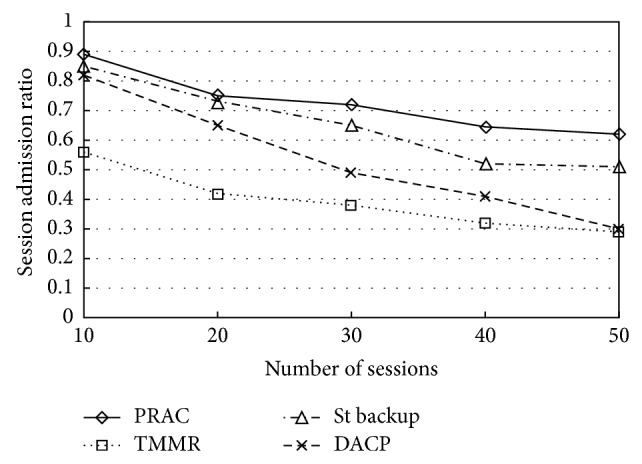
Session admission ratio.

In Figure 8 we inspect the number of sessions that each protocol permits to be active at the same time. It can be agreed that when the number of sessions is minimum, the variance among the protocols is negligible. However, when a larger number of sessions are initiated, PRAC is able to sustain that more than all other three protocols. This contributes to the variances in delivery rate among the protocols and proves that PRAC is capable of using the resources in the network more efficiently.

**Figure 9:**
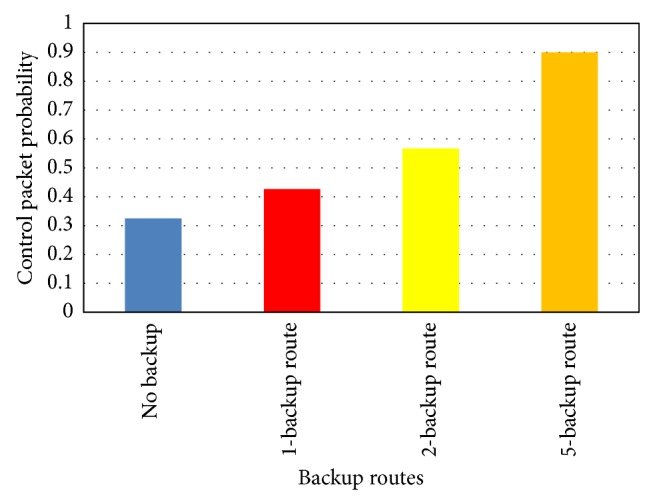
Identifying the overall control packet probability when using different backup path.

Finally, as shown in Figure 9 we compare different backup path approach to identify the overall control packet probability. It is obvious that the probability is least for no Backup approach, as there is no much of backup transmission planned after the failure attempt in first time. Also the trend in the shown diagram depicts that the higher the backup routes, the larger the control packet probability in the network. To balance the overhead or the control packet probability and to manage the overall data transmission quality, single backup path will be the better choice as it has least probability next to no backup approach but still we have a backup path to handle the earlier failure attempts thus making sure the continuous operation of data transmission.

## 5. Conclusion and Future Work

Pitching the performance of any network, keeping it uncompromised depends upon the QoS provided by the network. If that is the case, our proposed PRAC model minimizes the delay and maximized throughput for any admitted session thereby increasing the overall performance. However, the model considers the respective nodes capacity, analyzing its neighbor's capacity. The QoS provided eventually does not bring up any constrains on reliability. Considering our environment where mobility is high, our model ensures a tested backup path, which holds the key to revival from the path break. Providing many backup paths increases the control overheads, whereas PRAC considers only one tested backup path and this relatively reduces the control overheads. In general, in the mobile networks, resource reservation provides QoS. Our reservation procedure supports real time applications by avoiding the hidden and exposed terminal problems. Thus it provides collision free reservation and minimized delay. Our protocol PRAC, which includes admission control along with reservation mechanism, ensures much minimized delay. We attain a problem free reservation, but still connectivity poses a major problem in the mobile ad hoc network. In future, studies can be made on providing QOS without any connectivity issues, which paves way for many research enhancements.

## Figures and Tables

**Algorithm 1:**
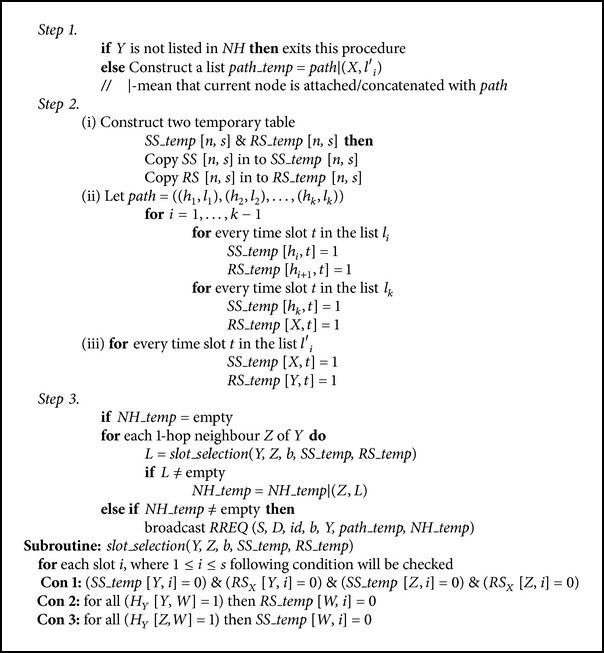


**Algorithm 2:**
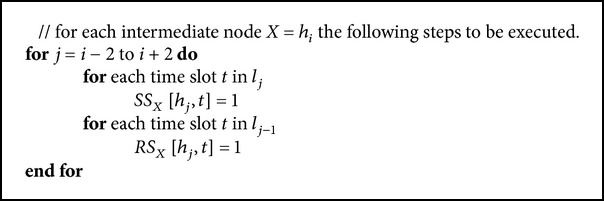


**Table 1:** Parameter settings.

Parameters	Values
Propagation model	Two-ray ground
Reception range	250 m
Carrier sensing range	550 m
Data packet size	512 bytes
CBR data rate	128 kbps
Network area	1000 m × 1000 m
Mobility model	Random way point
Backup route query (BRQ)	2 s
Channel bit rate	2 Mbps
Number of nodes	100
TDMA frame length	16 time slots
Slot time	5 ms
Simulation time	100 sec
Maximum number of sessions	50
